# Pain management in acute otitis media: a qualitative study of parents’ views and expectations

**DOI:** 10.1186/s12875-019-0908-9

**Published:** 2019-01-23

**Authors:** Rick T. van Uum, Roderick P. Venekamp, Anne G. M. Schilder, Roger A. M. J. Damoiseaux, Sibyl Anthierens

**Affiliations:** 1 Julius Center for Health Sciences and Primary Care, University Medical Center Utrecht, University Utrecht, Office number FAC 5.09, P.O. Box 85500, 3508 GA Utrecht, The Netherlands; 2 evidENT, Ear Institute, University College London, London, UK; 3 Department of Primary Care and Interdisciplinary Care, University of Antwerp, Antwerp, Belgium

**Keywords:** Acute otitis media, Children, Parents, Pain management, Primary care, Qualitative study

## Abstract

**Background:**

For unclarified reasons, parents tend to be cautious about administering analgesics to their children, potentially leading to suboptimal management of AOM symptoms. We aim to understand parents’ views and expectations of pain management in acute otitis media (AOM) in children.

**Methods:**

Qualitative study alongside a cluster-randomised controlled trial (PIM-POM study) aimed at optimising pain management in childhood AOM. We purposefully sampled 14 parents of children diagnosed with AOM by their GP, who were recruited to the trial between November 2017 and May 2018. Semi-structured interviews were held at home in the first two weeks after trial enrollment. Interviews were audio-recorded, transcribed and analyzed thematically.

**Results:**

Parents experienced difficulties in recognising earache and other symptoms of an ear infection. They consulted the GP for a diagnosis, for reassurance and for management advice. Parents shared that, prior to consultation, they had insufficient knowledge of the benefits of correctly dosed pain medication at regularly scheduled intervals. Parents valued the GP’s advice on pain management, and were happy to accept pain medication as standalone therapy, provided that the GP explained why antibiotics would not be needed. Parents’ views and expectations of pain management in AOM were shaped by previous experiences of AOM within their family; those with a positive experience of pain medication are more likely to use it in subsequent AOM episodes.

**Conclusions:**

Parents of children with AOM consult the GP to help cope with uncertainties in recognising symptoms of AOM, and to receive management advice. It is important that GPs are aware of parents’ lack of understanding of the role of pain medication in managing AOM, and that they address this during the consultation.

**Trial registration:**

Netherlands Trial Register, identifier NTR4920 (registration date: 19 December 2014).

**Electronic supplementary material:**

The online version of this article (10.1186/s12875-019-0908-9) contains supplementary material, which is available to authorized users.

## Background

Acute otitis media (AOM) is among the most common infectious diseases and reasons for doctor consultations and antibiotic prescribing in children [1–3]. Earache is central to children’s and parents’ experience of the condition [4, 5], Although it is widely accepted that this is best managed with analgesics that are dosed based upon weight [6, 7]; its best use is often mentioned casually rather than discussed specifically during consultations for AOM [8, 9], Importantly, parents tend to be cautious about administering analgesics to their children, due to concerns about adverse effects or a lack of clear instructions [10, 11], This may result in suboptimal management of AOM symptoms, in particular earache, as well as future reconsultation and antibiotic prescriptions.

Surprisingly little research has been done to understand parents’ views and expectations of pain management for AOM in children. Most strategies to improve AOM management so far have focused on reducing antibiotic prescriptions, targeting medical professionals [12, 13], thereby overlooking parents’ key role in decision making.

We therefore conducted a qualitative study alongside a trial that aimed to optimise pain management in children with AOM.

## Methods

In this process evaluation, we interviewed parents of children who participated in the trial (PIM-POM study). The trial was a primary care-based, cluster-randomised controlled trial of a multifaceted educational intervention that aimed to optimise pain management in children with GP-confirmed AOM and ear pain. In this trial, 37 Dutch GP practices were randomly allocated to either the intervention or control group. Prior to the first inclusion, GPs in the intervention group received an educational intervention consisting of an online training module and a face-to-face visit by the study physician. Both interventions aimed at educating GPs about pain management in AOM; encouraging them to use a parent information leaflet to discuss pain management in each AOM consultation and prescribe both paracetamol and ibuprofen according to current guidelines [14], rather than rely on over-the-counter (OTC) purchase of these medications. GPs in the control group did not receive this educational intervention and provided usual care. To ensure standardised AOM diagnosis, all participating GPs, in both intervention and control group, completed an online module illustrating various otoscopic images (i.e. normal appearance of tympanic membrane, AOM, and otitis media with effusion). The main outcome measure of the trial was the mean difference in ear pain score on Wong-Baker Faces Scale over the first three days [15]. Full details on the trial’s rationale and design have been published elsewhere [16]. The translated parent information leaflet is available in Additional file 1.

### Study population and setting

We asked parents of children, recruited to the trial between November 2017 and May 2018, to take part in this interview study. They were approached by phone within three days of enrollment. We purposefully sampled parents from both the intervention and control group, taking into account parents’ age, sex, education level, and employment status. We expected to conduct 10 to 20 semi-structured interviews to reach data sufficiency [5] [17].

### Data collection and analysis

After obtaining written informed consent, the study physician (RTvU) interviewed parents at their homes within two weeks of trial enrollment. Interviews were semi-structured, and focused on the management of their child’s current AOM episode, in particular pain management. The interview guide (see Table 1 and Additional file 1) was constructed through literature review and input of the expert research team. All interviews were audio-recorded and transcribed verbatim. Interviews were analyzed thematically in an iterative process [18, 19].

**Table 1:** Summary of the interview guide^a^

Topics	
*What are your views on earaches?*	
*How can you tell when your child has an earache?*	
*What are your greatest concerns?*	
*What do you do when your child has an earache?*	
*What reasons made you contact the GP (practice)?*	
*What did the GP discuss with you?*	
*Which aid did the GP use to support what he/she was telling you?*	
*Did the GP change your previously-held ideas/opinions about OMA and its treatment?*	
*How did the recent appointment differ from your previous visit(s)?*	
*Did the prescribed pain medication help?*	
*Has your participation in the study impacted your views on OMA and pain relief?*	

^a^The full topic list is available online in Additional file 2

R,TvU and SA (sociologist) reviewed the initial interview transcripts for accuracy and decided whether the interview guide needed adaptation. The same researchers analyzed and coded the first four interviews, line by line. Next, the open codes were grouped into bigger codes; categories with common meaning were grouped. Descriptions of each theme and sub-theme were added, along with quotes to support each. In order to ensure the clarity of the themes RTvU, SA, RV, RD discussed and interpreted these categories into potential themes. These themes were discussed within the team and reformulated at several stages during the analysis process. We collected data until saturation was reached.

## Results

Between November 2017 to May 2018, 18 GP practices across the Netherlands recruited 44 children with AOM to the PIM-POM study. We invited parents of 19 of these children to take part in this qualitative study; 25 parents were not actively approached for various reasons: not being able to reach parents by phone, insufficient time during phone call to obtain proper informed consent, or due to purposeful sampling (parents with similar characteristics already present in study sample). Of the invited parents, six parents declined due to time constraints and 14 parents of 13 children consented to be interviewed (mean interview duration was 19 min).

Table 2 summarises the parents’ characteristics; nine mothers and five fathers, with a range of educational backgrounds and employment status. Most parents had one or two children, had consulted the GP up to two times for their child’s current AOM episode, and half of the children attended daycare.

**Table 2:** Baseline characteristics

No	Gender	Age	Trial group	Education
1	Female	35–40	Control	Higher professional
2	Female	30–35	Intervention	Higher professional
3	Male	30–35	Intervention	University
4	Male	30–35	Intervention	Higher professional
5	Female^a^	25–30	Intervention	Vocational training
6	Male^a^	30–35		Vocational training
7	Female	35–40	Control	Vocational training
8	Female	40–45	Control	Higher professional
9	Female	35–40	Control	Higher professional
10	Male	30–35	Control	University
11	Female	30–35	Intervention	Vocational training
12	Female	25–30	Intervention	None
13	Male	40–45	Intervention	Vocational training
14	Female	30–35	Intervention	University

^a^Parents of the same child

Three themes developed from the analysis: reasons for consultation, empowerment of parents in managing AOM, and future AOM management.

### Reasons for consultation

#### Recognising AOM

Parents found it difficult to recognise symptoms of AOM in their child, in particular earache, and distinguishing AOM symptoms from those of other common childhood conditions, such as teething or the common cold. Some parents felt guilty for not recognising symptoms of AOM early on as they felt that their child suffered considerably.

*R2: “...and the trouble is that it takes a few days before I realise we are dealing with an ear infection. So by then, he [child] has been cranky for some time; before he develops an obvious fever and can express that he is in pain, we are at least two days on.”*

Parents’ awareness of their child’s suffering was shaped by their own experience of earache; parents who remembered how they themselves had suffered from earache as a child chose to seek medical care for their child urgently.

*R2: I have had AOM episodes as a child, I vividly recall them, each slight breeze of wind in my ear was as if someone stuck a knife in my ear [makes a stabbing gesture]. That is why I feel we should immediately act on it, together with the GP.*

Parents consulted the GP to receive a diagnosis, as they found it difficult to recognise AOM as the underlying cause of their child’s symptoms. Consultation was triggered by a range of symptoms, in particular fever, earache, sleep disturbance, general illness, and excessive crying. In other instances, parents consulted when they felt self-managing the illness had failed.

#### Reassurance

A comprehensive history and physical examination, in particular otoscopic examination, made parents’ feel that they were taken seriously and that they received the correct diagnosis and treatment.

*R9: “...that, whether she [GP] confirms it [ear infection] or not, at least she will have a look in his ears. That she takes a moment to look at her [child], just to see what is going on, so that I know what to do and expect. Because, obviously, I cannot say whether she has an ear infection or not. It is just as likely she has a tooth coming in, and... that certainty, really, is what I am after...”*

Apart from receiving a diagnosis, parents consult their GP to rule out severe illnesses or complications; they would rather consult ‘unnecessarily’, than fail to recognise a severe illness.

*R2: “…I feel like it is better to go [to the GP] ten times, and be told there is nothing wrong, or only something minor…than, uh, to have waited too long even once…”.*

#### Management advice

Parents sought advice on how to best manage their child’s symptoms, and felt their GP was the best person to give this information. They mostly wanted practical advice that provides rapid symptom relief.

*R7: …to be certain she actually has an ear infection, and to see whether it can be treated, to relieve her earache.*

### Empowerment of parents in managing AOM

#### Advice on analgesics in general

Many parents were unaware that AOM is usually self-limiting, neither were they aware of the importance and the potential benefits of pain medication, and how to best give these to their child.

*R2: “I am always cautious with pain medication and he [GP] really explained that – that there is no need for that […] I did not know that pain could have such a big impact on his [child] day, you know, and that you could relieve it so effectively with paracetamol.”*

Once parents felt they recognised earache or an ear infection, most initially self-managed this with treatments, such as olive oil ear drops, nasal saline or decongestant sprays, and paracetamol.

Many parents were cautious about giving paracetamol in the recommended dose and frequency; they would only give paracetamol when they felt it was absolutely necessary, and often dosed conservatively.

Parents who had received information from their GP about weight-based dosed paracetamol at regularly scheduled intervals (regardless of trial group) often had not realised, prior to consultation, that this regimen is safe and more effective. They appreciated advice on pain management and were happy to accept pain medication as standalone therapy, provided that their GP had performed a complete physical examination of their child, and had explained the natural course of AOM and why antibiotics were not needed.

*R2: “Well, I used to have the idea that pain medication was not enough, which is why, if things went on too long, I would ask for antibiotics on top of that. And now I see that pain medication alone is fine, that you still see improvement with only that.”*

Parents who had received information on weight-based dosing during the consultation expressed that they closely adhered to the GP’s recommendations afterwards.

*R3: “yes, we simply did provide, at first the maximum dosage and then after two days we adjusted to half the dosage [as was advised in the leaflet].”*

#### Advice on ibuprofen

In those cases where the GP advised parents to give their child ibuprofen in addition to paracetamol, it was revealed that parents were not aware that it was safe to give ibuprofen to children prior to the GP’s advice. Neither did they know about the OTC availability of ibuprofen. Parents attributed stronger analgesic properties to ibuprofen than to paracetamol. Those who gave ibuprofen to their children felt it was effective.

*R4: “…it [ibuprofen] worked extremely well. I felt it worked better than paracetamol, actually... I really thought so; with it, she [patient] was able to feel better for a few hours. And before, with paracetamol, we [parents] did things differently, too, because we were hesitant to give it during the day. It was more something for night-time, when she needed to sleep...”*

#### Discussing antibiotics

Parents were cautious about antibiotics, especially when prescribed repeatedly. When asked, parents referred to potential harmful effects of (repeated) antibiotic use such as antibiotic resistance. Participants felt that GPs rarely discuss the potential benefits and harms of antibiotics for AOM. Parents were happy to refrain from antibiotics when their GP carefully explained why antibiotics were unnecessary.

*R4: “...that it is true, you know, they do not always need antibiotics to get better. In the past, that had often been the case with her [child] ...”*

#### Supportive material

In general, parents felt that supportive material such as information leaflets, booklets or medical websites is useful. Parents in the intervention group valued the trial’s information leaflet and felt that it provided clear instructions on dosage and frequency of paracetamol and ibuprofen. The leaflet allowed them to recap the information at home. Parents valued GP’s recommendations regarding visiting websites that provide trusted information. Parents expressed having more faith in the information provided by their GPs than collected by themselves from other sources.

*R8: “Well, if you are familiar with it [ear infection], that means you have already Googled it, so I… but when I come home and I do not know exactly what is wrong with her [child], yeah, if you Google ‘earache’, well…you read some pretty shocking things. So then it is nice when he [GP] comes right out and says, go ahead and look it up…”.*

#### Prescriptions versus OTC use of analgesics

Parents (regardless of trial group) felt there was no need for a prescription of paracetamol or ibuprofen since these drugs are widely available OTC. They considered GPs’ instructions, together with those given by the pharmacist, sufficient. Certain parents, however, felt that a prescription for ibuprofen may, in general, lower the threshold for (other) parents to actually give this drug.

### Future AOM management

Parents who had a positive experience with pain medication had developed a greater self-efficacy in regards to self-management of their child’s AOM. They were more inclined to give these drugs in the appropriate dose if symptoms would recur. GP advice on the use of pain medication enhanced parents’ confidence in this particular management strategy, and made them more willing to initiate pain medication in subsequent episodes. Furthermore, they felt more confident to give higher and more regular doses of pain medication. Parents also reported that GP’s advice on ibuprofen had lowered their threshold to use this in the future.

*R12: “...I think that I would then [with subsequent ear infection] be more likely to stick to pain medication and try to get by without using antibiotics...”*

Nevertheless, some parents did not feel confident to manage future AOM episodes themselves and would re-consult their GP in the future to rule out potential underlying or serious illness that can only be identified by clinicians. This is particularly the case in situations where parents were previously faced with a severe illness in their child or when they had older children with similar or recurrent problems.

*R2: “I would probably handle it [future AOM episode] exactly as I did this time. So, first I would see the GP to find out if one or both ears are involved. And after that, yeah, try to get by on pain medication as much as possible.”*

## Discussion

### Main findings

Parents of children with AOM seek professional help to receive a diagnosis, reassurance and management advice. Parents value GP’s advice on the use of pain medication; they generally have insufficient knowledge of the role of pain medication in AOM. They gained new knowledge and accept pain medication as standalone therapy, provided that the GP explains why antibiotics would not be needed. Parents’ views and expectations of pain management in AOM are shaped by previous experiences of AOM within their family; those with a positive experience of pain medication are more likely to use it in subsequent episodes. Parents valued consultations that provided management advice on pain medication and reassurance when consulting for AOM, especially when antibiotics were not needed according to the GP.

### Comparison with existing literature

Few studies so far have explored parents’ views of AOM management, and these primarily focussed on the role of antibiotics [5] [20–22], Among a convenience sample of parents of children attending Australian day-care centres, Hansen et al. found that most parents assume that antibiotics were the best treatment option [5]. These parents were primarily higher-educated mothers. They all gave their children pain medication for temperature reduction or pain relief, but they considered these drugs alone insufficient to treat AOM. Meherali et al. found the same in purposive and convenience samples from a paediatric emergency department in Canada [22].

In contrast, we found that parents do accept pain medication as standalone treatment, when the GP has taken time to discuss why antibiotics are not needed. We also found that Dutch parents are cautious about using antibiotics, and would rather avoid them if possible. In the Netherlands, a watchful waiting strategy for AOM has been advocated for decades, and antibiotic prescription rates are among the lowest globally. Nevertheless, our findings are consistent with the findings among a purposive sample of parents from urban GP practices in the United Kingdom [20]. Parents in Australia, Canada, the UK, the Netherlands and Iceland [21] all seem aware of potential harmful effects of antibiotics, especially when used repeatedly.

Other qualitative studies of parents’ perceptions on symptom management have focused on acutely ill children or those with respiratory tract infections [23–31]. In line with our findings, these studies show that parents mainly consult their GP for diagnosis and for reassurance, and they do not want antibiotics when they will not relieve current symptoms.

The studies on parents’ views of AOM management confirm parents’ difficulties in recognising AOM as the underlying cause of their children’s distress, and their search for reassurance, as well as ways to relieve their child’s symptoms [5] [20, 21]. In general, parents value a discussion of management options by their GP [5] [20, 21].

Our study shows that parents generally seem to prefer a non-antibiotic prescribing strategy and accept pain medication as standalone therapy where possible. On the other hand, the GPs in our trial that were interviewed in a separate qualitative study tend to focus on treating the infection and whether or not to prescribe antibiotics for AOM [32]. This prescribing behaviour often results from perceived parental pressure or feelings of helplessness in managing symptoms with analgesics. Importantly, both parents and GPs are aware of the pain caused by of AOM, and strive to relieve the child’s symptoms. We believe there is a need for research identifying strategies on how to alleviate these symptoms most effectively, and recommend GPs to address pain management in AOM consultations.

### Strengths & limitations

Regular review and discussion of coding and analysis with all members of a multidisciplinary research team (researcher triangulation) has contributed to consistency in data synthesis and interpretation [33–35], as well as increased trustworthiness [36]. The different background of coders (sociologist and physician) enhances reliability and reflexivity and has helped to set aside any preconceptions [37].

One could argue that participants changed their behaviour due to trial participation, the so-called Hawthorne-effect, and that this may have influenced parents’ perceptions on AOM management. We aimed to minimise this issue by encouraging parents to provide their views and expectations regardless of any study procedures, and by including parents from the control group who were not aware of the intervention and received usual care.

Our study population only included parents who consulted their GP because their child suffered from AOM symptoms. As such, it is uncertain to what extent our findings can be transferred to the substantial number of parents who self-manage their child’s AOM episodes at home [38].

Campaigns and guidelines in the last decades in the Netherlands have emphasized the importance of adequate analgesia and judicious antibiotic use for upper respiratory tract infections in children, and antibiotic prescribing rates in general practice are among the lowest in the world [39]. Parents in our sample generally seem aware of this antibiotic policy, but not of the full range of analgesic possibilities. Our results may be transferrable to countries with comparable attitudes towards AOM management, regardless of who is primarily responsible for managing childhood AOM episodes (e.g. GP, primary care paediatrician, or nurse practitioner), under the condition that all healthcare professionals consistently provide parents with management options, that are in line with public campaigns and/or clinical practice guidelines [40, 41].

## Conclusion

In conclusion, parents of children with AOM consult their GP to help cope with uncertainties in recognising AOM and to receive management advice. It is important that GPs, and other clinicians involved in the care of children with AOM, are aware of parents’ lack of understanding of the role of pain medication in managing AOM and address this during the consultation, to better align with parents’ views and expectations.

## Additional files

Additional file 1: Translated parent information leaflet about pain relief for children with middle ear infection. To be used by the general practitioner as part of the intervention, to discuss pain management in each acute otitis media consultation (PDF 666 kb)

Additional file 2: Interview guide as used by the study physician as guidance for the semi-structured interviews. (DOCX 28 kb)

## Abbreviations

AOM: Acute otitis media
GP: General practitioner
OTC: Over-the-counter
