# Supplementary material for: Pain management in acute otitis media: a qualitative study of parents’ views and expectations
Source: BMC Fam Pract. 2019 Jan 23;20:18. doi: 10.1186/s12875-019-0908-9 (PMC6343236; doi:10.1186/s12875-019-0908-9)
Supplement: Supplementary file 1 — Translated parent information leaflet about pain relief for children with middle ear infection. To be used by the general practitioner as part of the intervention, to discuss pain management in each acute otitis media consultation (PDF 666 kb) [file 12875_2019_908_MOESM1_ESM.pdf]

Schedule 3 Ibuprofen (20mg/kg/day), **max** 3 days. Give **either** syrup **or** tablets **or** suppositories

Tick:	Weight of child in kg	Syrup (20 mg/ml)	Tablets (in the mouth)	Suppositories (in the bottom)
	9-11	3-4 times 2 ml		
	12-15	3-4 times 3 ml		
	16-19	3-4 times 4 ml		
	20-23	3-4 times 5 ml	3-4 times 1 tablet of 100 mg	
	24 kg	3-4 times 6 ml	3-4 times 1 tablet of 100 mg	
	25-29		3-4 times 1 tablet of 100 mg	3-4 times 1 supp of 125 mg
	30-39		3-4 times 1.5 tablet of 100 mg	3-4 times 1 supp of 125 mg
	40-49		3-4 times 1 tablet of 200 mg	3-4 times 1 supp of 125 mg

supp: suppository

If your child doesn't recover despite the treatment

If you are worried, because your son or daughter doesn't recover despite the treatment, feels drowsy or doesn’t drink well, or if the earache gets worse or isn't gone after three days, you should contact your GP again.

Finally

More information about middle ear infection can be found on www.thuisarts.nl. The dosages for children in this leaflet are taken from the pharmacotherapeutic guideline for pain control (*‘Farmacotherapeutische richtlijn Pijnbestrijding'*) published by the Dutch College of General Practitioners (*NHG)*. This leaflet is not a package insert. You can obtain more information about the medication at your pharmacy, or read more at www.consumed.nl.

UMC Utrecht

PIM-POM study team
Prof. Roger Damoiseaux PhD, general practitioner, Prof. Anne Schilder PhD, ENT specialist, Roderick Venekamp PhD, general practitioner, and Rick van Uum MD, GP in training

© The Author(s). 2018 This leaflet was originally published in Trials under the terms of the Creative Commons Attribution 4.0 International License (http://creativecommons.org/licenses/by/4.0/), which permits unrestricted use, distribution, and reproduction in any medium, provided you give appropriate credit to the original author(s) and the source, provide a link to the Creative Commons license, and indicate if changes were made.

PIM-POM STUDY

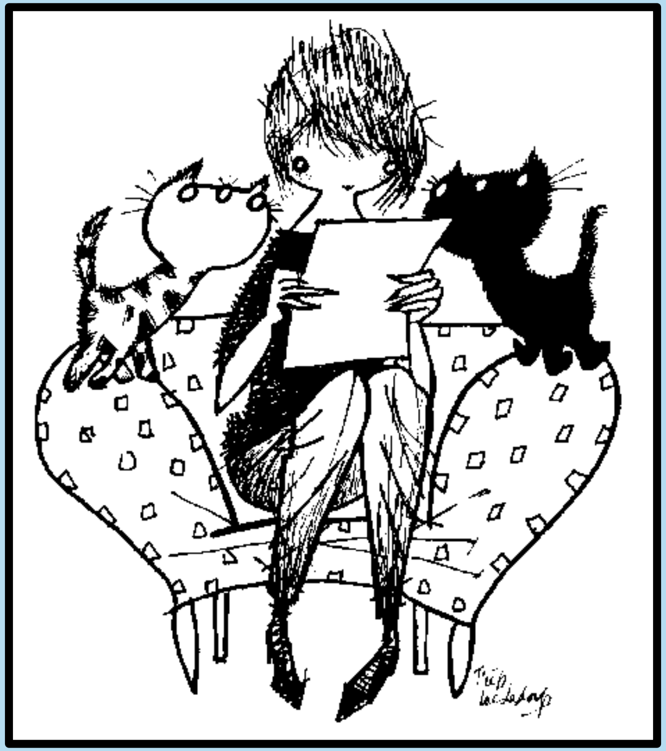

©Fiep Amsterdam bv; Fiep Westendorp Illustrations. These are the cats Pim and Pom. For more information, visit the website: www.pimenpom.nl

Information leaflet about pain relief for children with middle ear infection

Introduction

Your GP has diagnosed your son or daughter with a middle ear infection. A middle ear infection can be very painful. The worst symptoms usually subside in two to three days. In children under the age of two, symptoms can last up to a week. The GP has given you a prescription for pain medication. In this leaflet we explain how best to give this pain medication to your child.

Paracetamol

Paracetamol alleviates the earache and suppresses the fever. It will start to work after 15 to 30 minutes, and will have an effect for 3 to 5 hours. Please wait at least 4 hours before giving the next dose of paracetamol, and at least 6 hours when you use a paracetamol suppository. Paracetamol is available as a tablet, syrup or suppository. Tablets or syrup are preferred, because paracetamol is absorbed better that way. In the first three days of the middle ear infection, it's important to give paracetamol to your child in a high dosage, following schedule 1. After three days, you decrease the dosage of paracetamol by switching to schedule 2.

Ibuprofen

For children older than 1 year with earache despite paracetamol according to schedule 1 or 2, ibuprofen may additionally be offered. Ibuprofen is stronger pain medication, which reduces the inflammation of the ear and suppresses the fever. It will start to work after 30 to 60 minutes and will have an effect for 8 hours. Wait at least 6 hours before giving the next dose of ibuprofen. Ibuprofen should **never** be given for more than three consecutive days. You will find the dosage of ibuprofen on the next page in schedule 3.

Facts about pain medicine

It's important that you give your child pain medication regularly, as instructed. This way, the amount of pain medication in the blood remains stable and the pain is reduced evenly. So don't wait until your child has an earache again, but give the next dose in time. This will also help to prevent your child waking up at night because of the pain. During the first three days, give the standard dose of paracetamol 4 to 6 times a day as a syrup **or** using tablets, **or** give a suppository 3 times a day (see schedule 1). Then gradually decrease the dosage.

© The Author(s). 2018 This leaflet was originally published in Trials under the terms of the Creative Commons Attribution 4.0 International License (http://creativecommons.org/licenses/by/4.0/), which permits unrestricted use, distribution, and reproduction in any medium, provided you give appropriate credit to the original author(s) and the source, provide a link to the Creative Commons license, and indicate if changes were made.

Myths about pain medicine

- *'If I give painkillers too often, it will be less effective.'* This is **not** true. Your child cannot become 'insensitive' to pain medicines and they are not addictive.
- *'I will save the pain medication for bedtime in the evening, that's when my child really needs it.'* It's true that in most children the earache worsens in the evening; in the dark there's less distraction so the pain is more notable. But it's just as important to alleviate the pain during the day.

Schedule 1 Paracetamol (90mg/kg/day), **max** 3 days. Give **either** syrup **or** tablets **or** suppositories

Tick:	Weight of child in kg	Syrup (24 mg/ml)	Tablets (in the mouth)	Suppositories (in the bottom)
	7	4-6 times 4 ml		3 times 1 suppository of 120 mg
	8-9	4-6 times 5 ml		3 times 1 suppository of 240 mg
	10-11	4-6 times 6 ml		3 times 1 suppository of 240 mg
	12	4-6 times 7 ml		3 times 1 suppository of 240 mg
	13-14	4-6 times 8 ml		3 times 1 suppository of 240 mg
	15	4-6 times 9 ml	4-6 times 1.5 tablet of 120 mg	3 times 1 suppository of 240 mg
	16	4-6 times 10 ml	4-6 times 1.5 tablet of 120 mg	3 times 1 suppository of 240 mg
	17	4-6 times 10 ml	4-6 times 1 tablet of 250 mg	3 times 1 suppository of 500 mg
	18-19	4-6 times 11 ml	4-6 times 1 tablet of 250 mg	3 times 1 suppository of 500 mg
	20-24		4-6 times 1 tablet of 250 mg	3 times 1 suppository of 500 mg
	25-33		4-6 times 1.5 tablet of 250 mg	3 times 1 suppository of 500 mg
	34-49		4-6 times 1 tablet of 500 mg	3 times 1 suppository of 1000 mg

Schedule 2 Paracetamol (60mg/kg/day), **after** 3 days. Give **either** syrup **or** tablets **or** suppositories

Tick:	Weight of child in kg	Syrup (24 mg/ml)	Tablets (in the mouth)	Suppositories (in the bottom)
	7	4-6 times 2 ml		3 times 1 suppository of 120 mg
	8-9	4-6 times 3 ml		3 times 1 suppository of 120 mg
	10-11	4-6 times 4 ml		3 times 1 suppository of 120 mg
	12-14	4-6 times 5 ml		3 times 1 suppository of 240 mg
	15-16	4-6 times 6 ml	4-6 times 1.5 tablet of 100 mg	3 times 1 suppository of 240 mg
	17-19	4-6 times 7 ml	4-6 times 1.5 tablet of 100 mg	3 times 1 suppository of 240 mg
	20-21	4-6 times 8 ml	4-6 times 1.5 tablet of 100 mg	3 times 1 suppository of 240 mg
	22-23	4-6 times 9 ml	4-6 times 1.5 tablet of 100 mg	3 times 1 suppository of 240 mg
	24	4-6 times 10 ml	4-6 times 1.5 tablet of 100 mg	3 times 1 suppository of 240 mg
	25-37		4-6 times 1 tablet of 250 mg	3 times 1 suppository of 500 mg
	38-49		4-6 times 1.5 tablet of 250 mg	3 times 1 suppository of 500 mg
