# Supplementary material for: Pain management in acute otitis media: a qualitative study of parents’ views and expectations
Source: BMC Fam Pract. 2019 Jan 23;20:18. doi: 10.1186/s12875-019-0908-9 (PMC6343236; doi:10.1186/s12875-019-0908-9)
Supplement: Supplementary file 2 — Interview guide as used by the study physician as guidance for the semi-structured interviews. (DOCX 28 kb) [file 12875_2019_908_MOESM2_ESM.docx]

**Interview guide: process evaluation with parents (PIM-POM study)**

*Our sincere thanks to you for participating in this component of the PIM-POM study. The purpose of this interview is to learn how you perceived the appointments with your general practitioner (GP) for an acute middle-ear infection (AOM); what role the GP played in those perceptions; and what your current ideas and views are with regard to earaches, OMA and its treatment (specifically pain medication). This interview study will help us explore which factors may be vital to improving the treatment of AOM and better alleviating the associated pain.*

*While I will be recording the interview simply so that I do not have to write everything down, I would like to emphasise that everything recorded here will be treated as confidential. This recording and the resulting interview transcripts will be stored securely and anything that is said will be rendered anonymous and impossible to trace back to you. In other words: if information from your interview is used in articles or presentations, it will not be personally identifiable.*

*Do you have any questions before we begin the interview?*

Date:

Age of participant:

Gender:

Ethnicity:

Highest level of education attained:

Current job:

City and region of residence:

Number of children in the family:

The child attends day-care (Y/N):

Number of GP contacts for current AOM episode:

Prescribed medication and/or over-the-counter (OTC) or home remedy:

**Perspective on earache**

- What are your views on earache?
- How can you tell when your child has an earache?
- If your child has an earache, at what point do you feel worried? What are your greatest concerns?
- What do you do when your child has an earache? Does the severity of their pain affect your actions? Do you give an OTC or home remedy, and if so, which one?

**OMA consultation**

- Where did you get the idea to contact your GP: was it your own initiative, or the advice of the day-care, or from another source?
- What reason or reasons made (or would make) you decide to contact the GP's practice when your child has an earache?
  - *Follow-up questions in case this fails to come up naturally:*
    - What are your expectations for this contact?
- What reason or reasons made (or would make) you decide to schedule an appointment with the GP when your child has an earache?
  - *Follow-up questions in case this fails to come up naturally:*
    - During such an appointment, what do you expect from the GP?
- Did the recent appointment meet your expectations? Why or why not?
  - *Follow-up questions in case this fails to come up naturally:*
    - How did you feel at the end of the recent appointment? (Reassured, taken seriously, and so on)
    - What could the GP do to make sure you are satisfied when you leave his/her practice?
- What did the GP discuss with you?
  - *Follow-up questions in case this fails to come up naturally:*
    - Did the GP discuss the natural disease progression of AOM with you?
    - Did the GP speak with you about antibiotics? If so, what did he or she say?
    - Did the GP speak with you about pain medication? If so, what did he or she say?
    - Did the GP speak with you about potential symptoms that would be cause for alarm or reasons to contact the practice again?
    - Is there anything that was not addressed that you would have liked to hear or discuss?
- Were you able to form a clear understanding of everything the GP said to you? How would you summarise what you heard?
- During the appointment, did the GP use any aids (such as a website or information leaflet) to support what he/she was telling you? If so, how would you summarise that information?
  - *Follow-up questions in case this fails to come up naturally:*
    - In your opinion, how were those aids valuable (or not)?
    - Which aids helped you to better understand what the doctor was saying?
- Did the GP change your previously-held ideas/opinions about AOM and its treatment? If so, can you explain why?
  - *Follow-up questions in case this fails to come up naturally:*
    - Has your opinion regarding the use of antibiotics for AOM changed?
    - Has your opinion regarding the use of pain medication for AOM changed?
    - Which GP-related factors led to a change in your opinions with regard to these topics?
- To your mind, does it matter whether the GP verbally recommends certain medication (such as paracetamol) or writes you a prescription for it? If so, in what sense?
- In the event you had previously taken your child to see the GP for an earache: how did the recent appointment (the one after you began taking part in the PIM-POM study) differ from your previous visit(s)?
  - *Follow-up question in case this fails to come up naturally:*
    - What is your overall opinion of the care provided by your GP(s) for your child's OMA?
- What would you do if your child were to develop a similar infection in the future?

Overall perspective

- Did the prescribed pain medication help? If so, how could you tell?
- Has your participation in the study impacted your views on AOM and pain relief? If so, in what way?
- Are there any other topics that have not been mentioned here but that you'd like to say something about?

*Thank you again for your time!*
